# Isolated Solitary Intramedullary Spinal Cord Metastasis Presenting as the First Manifestation of Small-Cell Lung Cancer: Report of a Rare Case

**DOI:** 10.1155/2012/617280

**Published:** 2012-12-25

**Authors:** Yusuf Kurtuluş Duransoy, Mesut Mete, Mehmet Selçuki, Aydın Işisağ

**Affiliations:** ^1^Department of Neurosurgery, Faculty of Medicine, Celal Bayar University, 45040 Manisa, Turkey; ^2^Department of Pathology, Faculty of Medicine, Celal Bayar University, 45040 Manisa, Turkey

## Abstract

*Background*. Intramedullary spinal cord metastases presenting as the first manifestation of malignancies are extremely rare lesions. *Case Description*. The authors report a 74-year-old woman who presented with an isolated intramedullary spinal cord metastasis which presents as first manifestation of malignancy without central nervous system and/or other organ involvement. She went under surgery, and after histopathological evaluation, primary focus was determined in lung in positron emission tomography. She is still alive after 9 months since the first diagnosis of primary focus. *Conclusion*. In patients with solitary intramedullary lesion in the spinal magnetic resonance imaging, whole-body investigation might help for diagnosis of primary focus and approach to treatment.

## 1. Introduction

Intramedullary spinal cord metastasis (ISCM) represents 4.2%–8.5% of central nervous system (CNS) and clinically affects only 0.1%–0.4% of all cancer patients [1, 2]. Lung and breast carcinomas are the most frequent sources of ISCM [1, 2]. Melanoma, malignant lymphoma, colon carcinoma, ovarian carcinoma, and renal cell carcinomas are other original sites for metastasis to ISCM [3]. The clinical features of ISCM have been described as pain, neurological deficitis and autonomic dysfunction [1–4]. In this study, we present a case of a 74-year-old woman with single spinal intramedullary metastasis as the first manifestation of small-cell lung carcinoma. 

## 2. Case Report

**Figure 1:**
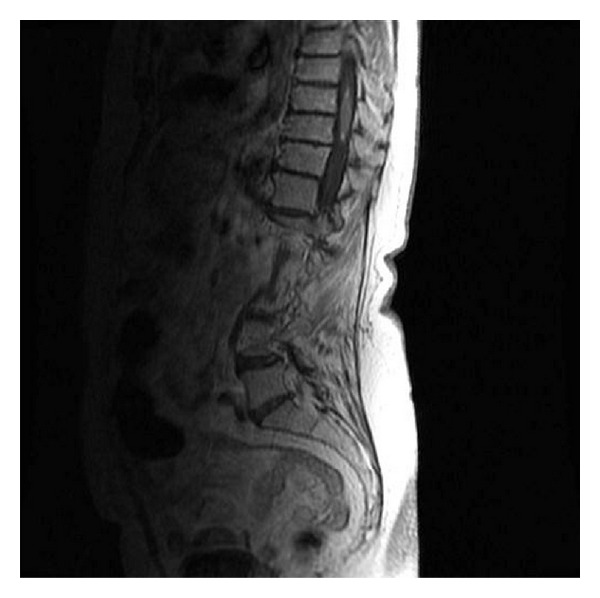
Thoracic MRI demonstrated a solitary intramedullary lesion at thoracic 9-10 level with homogeneous contrast enhancement in sagittal reconstruction.

 A 74-year-old woman admitted to neurosurgery department with a complaint of pain and numbness in both legs. These symptoms all had started 4 weeks earlier. Three weeks after onset of these first symptoms, she also experienced a loss of strength in both legs. On neurological examination, she had paraparesis. Hypoesthesia was demonstrated on the both sides below the level of thoracic 10. She had no fecal and urinary incontinence. She has been smoking for 45 years of 1 pack daily. Thoracic magnetic resonance imaging (MRI) of spinal cord demonstrated a solitary intramedullary lesion at thoracic 9-10 level with homogeneous contrast enhancement in sagittal reconstruction (Figure 1).

**Figure 2:**
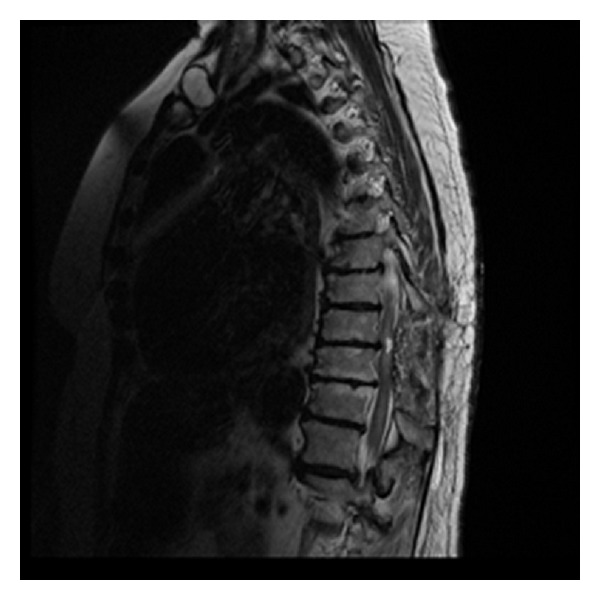
Postoperative sagittal thoracic MRI demonstrated total resection of ISCM.

Our differential diagnosis included neoplasm such as ependymoma, astrocytoma, intramedullary lymphoma, and other infectious diseases. In preoperative period craniospinal axis MRIs and chest X-ray were normal. Tumour was resected radically after dorsal midline myelotomy. Postoperative thoracic MRI demonstrated total resection of ISCM (Figure 2).

**Figure 3:**
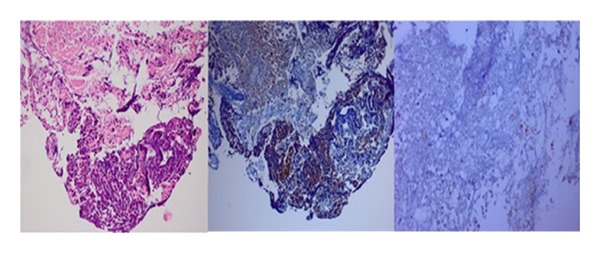
Malignant tumor consisting of small, carrot-shaped blue cells invading the surrounding soft tissue (H&E, ×100). Pan-cytokeratin immunopositivity in most of the tumor cells (DAB, ×100). Synaptophysin immunopositivity in a few necrotic tumor cells (DAB, ×200).

Histopathological report examination with hematoxylin eosin (H&E) revealed a malignant tumor consisting of small, carrot-shaped blue cells with widespread crushing artefact and invading the surrounding soft tissue. Metastatic epithelial nature of the tumor was shown with pan-cytokeratin staining in most of the tumoral cells while neuroendocrine differentiation was demonstrated with synaptophysin in a few necrotic tumoral cells (Figure 3). With these findings a diagnosis of small cell neuroendocrine carcinoma, probably metastatic from the lung, was done.

**Figure 4:**
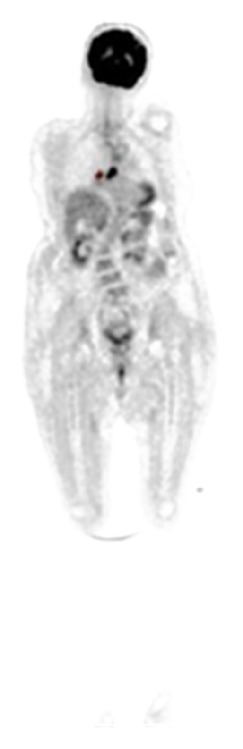
Positron emission tomography demonstrated a primary focus in lung.

Finally, patient was examined by whole-body positron emission tomography (PET) which showed a primary focus in lung without CNS and/or additional systemic metastasis (Figure 4).

Postoperative period was uneventful. There was no progression and regression at motor power of both legs. Chemotherapy was applied and referred to physiotherapy. She is still alive, for 9 months, from the first diagnosis of primary focus.

## 3. Discussion

Intramedullary spinal cord metastasis compromises only 1–3% of all intramedullary spinal cord neoplasms [5]. Lung (especially small-cell carcinoma) 29–54%, breast 11–14%, kidney 6–9%, colorectal 3–5%, melanoma 6–9%, lymphoma 4%, thyroid 2%, ovarian 1%, and approximately 3% were unknown primary that metastases to intramedullary spinal cord. ISCMs are very uncommon, and there are no imaging features that distinguish them from primary intramedullary tumours and paraneoplastic necrotising myelopathy in patients with previously known cancer focus [2]. The patient underwent thorough preoperative evaluation to exclude demyelinating, infectious, inflammatory, and other etiologies for the spinal cord mass such as ependymoma, astrocytoma, and intramedullary lymphoma, but there was no suspicion of ISCM because it was a solitary lesion, and there were no other signs or symptoms of malignancy. All radiological examinations were normal and finally we performed surgery for definitive diagnosis for subsequent treatment.

Schijns et al. reviewed the literature and reported that 55% of patients with ISCM had systemic metastases and 41% had brain metastases [3]. Sung et al. reported brain and systemic metastases as in 61% and 64% of ISCM patients, respectively, in their series [2]. Dam-Hieu et al. reported that 42% of patients had systemic and/or CNS metastases at the time of ISCM diagnosis in their study [1]. The output appears of ISCM as the first presentation of malignancy is occurred in 22.5% to 39% of ISCM cases [6]. It is very rare to find an isolated ISCM which presents as first manifestation of malignancy without central nervous system and other organ involvement in the literature. Donovan and Freeman reported a 41-year-old woman who had ISCM due to renal cell carcinoma without any metastasis to CNS and/or other organs [7]. Grasso et al. reported a 61-year-old woman who had ISCM due to metastatic colon cancer without CNS metastasis. Authors did not describe the presence of systemic metastasis [8]. In both studies, ISCM presented as the first manifestation of primary tumor [5, 8]. Our patient examined with craniospinal axis MRI and chest X-ray in preoperative period. As a result of pathological examination, whole-body PET showed primary focus at lung and there was no CNS and/or systemic metastasis.

Spreading of primary focus to intramedullary region might be via three pathways. (1) Hematogenous spread, (2) direct intramedullary invasion by meningeal carcinomatosis, and (3) direct invasion from contiguous structure [9]. There are different opinions in the literature for the localization of ISCM. Connoly reported that ISCM affected the cervical, thoracic, and lumbar cord equally [1, 2]. In a study of Kalayci et al. 147 patients with ISCM were analyzed and cervical cord was the most common site for metastases which might due to richer vascular supply [5]. Dam-Hieu et al. demonstrated that the most common site for ISCM was conus medullaris in their retrospective study with 19 patients [1]. Our patient's tumor was localized at thoracic 9-10 level.

Metastatic intramedullary spinal cord tumors cause pain, sensory disturbance, weakness, and sphincter dysfunction due to edema, distortion and compression of the spinal cord parenchyma [2]. Despite deterioration of neurological status is relatively rapid within a period of days to weeks in ISCM, symptoms typically present a slow progression in primary intramedullary tumors [2, 3]. Schijns et al. described a 70-year-old female who developed Brown-Sequard syndrome 6 weeks before presentation with an intramedullary spinal cord metastasis of an occult renal cell carcinoma [3]. Aryan et al. reported a 59-year-old male ISCM of a lung carcinoma who presented with decreasing of temperature sensation at right lower extremity and weakness at left lower extremity with 6 days history [4]. Our patient's symptoms had started 4 weeks before being admitted to us and deterioration of neurological status was rapid.

The mainstay of treatment for intramedullary spinal cord metastases in presence of previously known malignant disease is still unclear. There is no well-designed prospective clinical study comparing surgery and other therapy modalities such as radiotherapy and chemotherapy when administered alone or in combination due to rarity of intramedullary spinal cord metastasis [1–3]. Sung et al. reported that surgery in selected patients could result in the significant improvement of neurological function [2]. Also Dam-Hieu et al. operated 13 of 19 patients with ISCM and reported that surgery could result in significant improvement of neurological function [1]. Kalayci et al. reviewed and found that 33 of 138 patients operated in the literature had ISCM and postoperatively neurological improvement was observed in 66% of patients. Median survivals were 9.4 months and 5 months after surgery and conservative treatment, respectively [5]. In our department, the first treatment option for intramedullary spinal cord tumor is radical microsurgical removal of the tumor and additional treatment of radiotherapy and/or chemotherapy according to the result of histopathological report. After surgical removal, chemotherapy was applied and she was referred to physiotherapy.

## 4. Conclusion

 ISCM should be kept in mind in the presence of rapid progression of neurological deterioration and a solitary intramedullary lesion in the spinal MRI. In such cases without history of known malignant disease, preoperative whole-body investigation may help for diagnosis of primary focus. In the presence of solitary intramedullary lesion only in order to apply the appropriate therapy surgical removal should be applied to have a leading histopathological report.
